# A Community-Led Approach as a Guide to Overcome Challenges for Therapy Research in Congenital Disorders of Glycosylation

**DOI:** 10.3390/ijerph19116829

**Published:** 2022-06-02

**Authors:** Rita Francisco, Sandra Brasil, Carlota Pascoal, Andrew C. Edmondson, Jaak Jaeken, Paula A. Videira, Cláudia de Freitas, Vanessa dos Reis Ferreira, Dorinda Marques-da-Silva

**Affiliations:** 1 CDG & Allies—Professionals and Patient Associations International Network (CDG & Allies-PPAIN), Department of Life Sciences, School of Science and Technology, NOVA University Lisbon, 2819-516 Caparica, Portugal; rab.francisco@campus.fct.unl.pt (R.F.); sd.brasil@fct.unl.pt (S.B.); cm.pascoal@campus.fct.unl.pt (C.P.); andy.edmondson@gmail.com (A.C.E.); jaak.jaeken@kuleuven.be (J.J.); p.videira@fct.unl.pt (P.A.V.); 2 UCIBIO, Department of Life Sciences, NOVA School of Science and Technology, NOVA University of Lisbon, 2819-516 Caparica, Portugal; 3 Associate Laboratory i4HB—Institute for Health and Bioeconomy, School of Science and Technology, NOVA University Lisbon, 2819-516 Caparica, Portugal; 4 Portuguese Association for Congenital Disorders of Glycosylation (CDG), Department of Life Sciences, School of Science and Technology, NOVA University Lisbon, 2819-516 Caparica, Portugal; 5 Section of Biochemical Genetics, Division of Human Genetics, Department of Pediatrics, Children’s Hospital of Philadelphia, Philadelphia, PA 19104, USA; 6 Center for Metabolic Diseases, Department of Pediatrics, KU Leuven, 3000 Leuven, Belgium; 7 EPI Unit—Instituto de Saúde Pública, Laboratório para a Investigação Integrativa e Translacional em Saúde Populacional (ITR), Faculdade de Medicina, Departamento de Ciências da Saúde Pública e Forenses e Educação Médica, Universidade do Porto, 4050-600 Porto, Portugal; claudia_defreitas@yahoo.com; 8 LSRE-LCM—Laboratory of Separation and Reaction Engineering—Laboratory of Catalysis and Materials, Escola Superior de Tecnologia e Gestão, Instituto Politécnico de Leiria, 2411-901 Leiria, Portugal; 9 ALiCE—Associate Laboratory in Chemical Engineering, Faculty of Engineering, University of Porto, 4200-465 Porto, Portugal

**Keywords:** Congenital Disorder(s) of Glycosylation (CDG), drug development, mixed methods research, rare diseases, therapies, think tanks

## Abstract

Congenital Disorders of Glycosylation (CDG) are a large family of rare genetic diseases for which effective therapies are almost nonexistent. To better understand the reasons behind this, to analyze ongoing therapy research and development (R&D) for CDG, and to provide future guidance, a community-led mixed methods approach was organized during the 4th World Conference on CDG for Families and Professionals. In the quantitative phase, electronic surveys pointed to the prioritization of six therapeutic R&D tools, namely biobanks, registries, biomarkers, disease models, natural history studies, and clinical trials. Subsequently, in the qualitative phase, the challenges and solutions associated with these research tools were explored through community-driven think tanks. The multiple challenges and solutions identified administrative/regulatory, communication, financial, technical, and biological issues, which are directly related to three fundamental aspects of therapy R&D, namely data, sample, and patient management. An interdependence was traced between the prioritized tools, with diagnosis and therapies acting as bidirectional triggers that fuel these interrelationships. In conclusion, this study’s pioneering and adaptable community-led methodology identified several CDG therapy R&D gaps, many common to other rare diseases, without easy solutions. However, the strong proactive attitude towards research, based on inclusive and international partnerships and involving all members of the CDG community, sets the direction for better future therapy R&D.

## 1. Introduction

Congenital Disorders of Glycosylation (CDG) are a growing family of genetic diseases first described in 1980 [1]. Some 160 CDG and over 200 CDG phenotypes are currently known, with PMM2-CDG being the most prevalent [2,3]. CDG are clinically and biologically diverse, mainly presenting with predominant neurological involvement, accompanied by multi-organ impairment, among which are the eyes, the liver, the heart and the immune system [4,5,6,7]. Unfortunately, effective treatments are available for very few CDG. Current therapy options are mostly limited to symptom-management approaches, monosaccharide supplementation, and repurposed drugs [8,9,10,11].

Therapy research and development (R&D) encompasses several stepwise phases, from early drug discovery to post-market authorization and monitoring. Multiple tools and studies are integrated with these phases, ranging from drug/compound to biological material and clinical data repositories, disease models, and biomarkers. In addition, natural history studies (NHS) also play a critical role in therapy development. Overall, therapy R&D entails the identification of promising molecules and understanding of the pathophysiological mechanisms responsible for clinical phenotypes and disease evolution, culminating in controlled human testing in clinical trials (CTs).

On the one hand, CDG therapeutic research has been rapidly expanding in recent years [8,9,10,11,12]. There are currently a total of six ongoing observational studies (NCT04201067, NCT02089789, NCT04198987, and NCT03404856), including two NHS (NCT03173300 and NCT01417533), and four therapeutic CTs (NCT04833322, NCT04679389, NCT03404869, and NCT03404856). In parallel, significant advances in disease model development and biomarker discovery have also occurred [8,13,14,15,16]. On the other hand, CDG therapy-driven biomedical and clinical research is anticipated to be hindered by several challenges, many common to other rare diseases [17,18]. However, no study has investigated or described them. Additionally, the perceptions, experiences, and expectations of the CDG community, including families, researchers, and healthcare professionals, regarding therapy R&D are barely known.

Multi-stakeholder, community-led research exercises allow for a broader understanding of existing challenges and enable the proposal of solutions that can benefit all interested parties. These exercises present several advantages, including the improvement of decision quality, credibility, and implementation, as well as the promotion of cross-sector relationships and community empowerment [19].

Mixed methods research combines quantitative and qualitative data collection approaches that are especially suited to respond to complex questions involving multiple stakeholders. In rare diseases, mixed methods are already being used in therapeutic research to assess patients’ health needs and design rigorous patient-centric clinical and translational research [20,21,22].

In this study, a community-led, participatory mixed-methods research design was adopted to holistically explore the perceptions and experiences of the CDG community regarding ongoing and future biomedical and clinical research aimed toward therapy development. Members of the CDG community included healthcare professionals (HCPs), researchers, pharmaceutical industry representatives, and families. The main aim of this work was to develop, test, and implement an innovative, inclusive, and reproducible (potentially adaptable to other diseases and biological questions) mixed-methods approach that captures and integrates the research-related needs and perspectives of multiple CDG community stakeholders in order to improve the understanding of the current CDG therapy R&D landscape and guide future research efforts. To effectively achieve this aim, (1) an electronic survey (e-survey) was used to assess the importance attributed by the CDG community to different therapeutic research tools and to establish priorities; and (2) multi-stakeholder think tank discussions were subsequently held to further explore the quantitative findings and to identify challenges and solutions associated with the prioritized therapeutic research tools.

Under the scope of this special issue, we believe this work describes a highly valuable approach designed under the concept of patient-centered care that is replicable to any rare disease.

## 2. Methods

This mixed-methods research study was based on a sequential explanatory design, which used quantitative e-survey-derived data as a preliminary data source. Subsequently, qualitative data was gathered through think tank discussions to further interpret and clarify the results from the quantitative data analysis.

### 2.1. Quantitative Study

**Figure 1:**
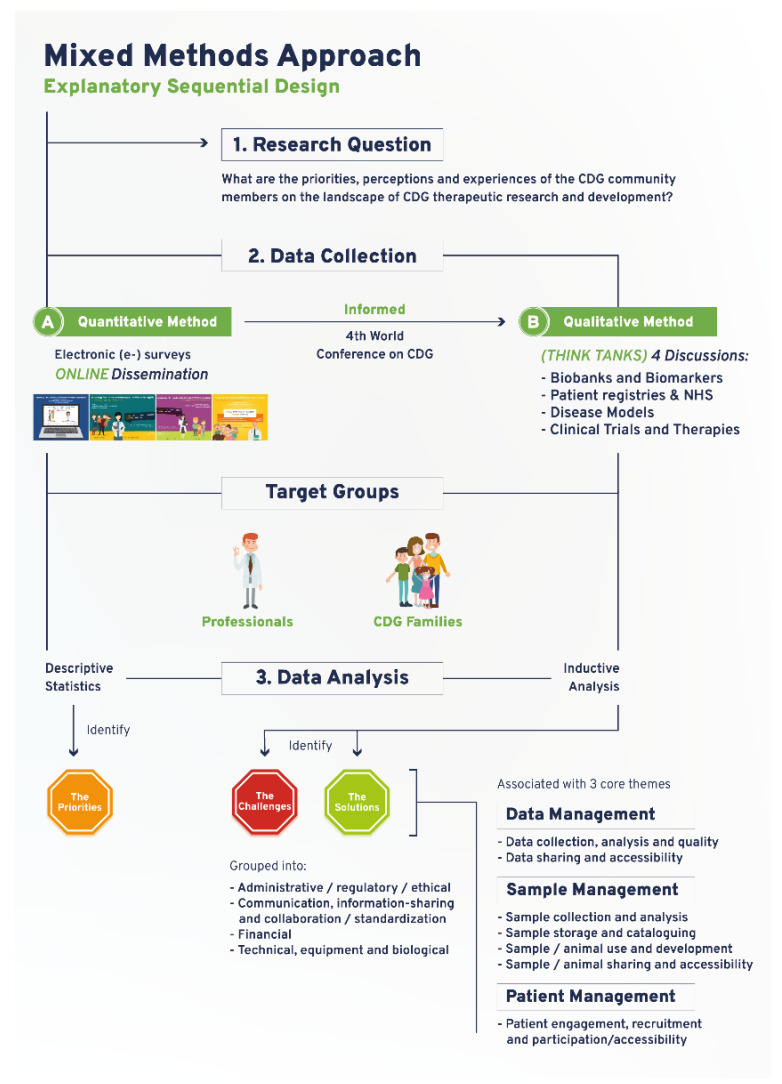
Schematic depiction of the devised and adopted explanatory sequential mixed methods research approach.

The e-survey was carried out to assess the views of CDG community members about therapeutic R&D (Figure 1). The original e-survey included a total of 68 questions and was available in English. This study draws on the results from 13 questions purposefully selected to identify priorities related to CDG therapy R&D tools. The questions were adapted to the two target populations: (1) CDG professionals, and (2) CDG families. A total of 128 respondents (82 family members of people living with CDG and 45 professionals), mostly from Europe (21 different European countries were represented) and the USA, participated in this study (Table S1). Ethical approval for this study was obtained from the ethics committee of the Faculty of Psychology, University of Lisbon (reference 1.14/8/2018-19). Electronic informed consent was obtained from all participants.

The e-survey was administered through the SurveyMonkey platform (http://www.surveymonkey.net, Copyright#1999–2019 SurveyMonkey). The e-surveys were launched online on 16 May 2019 and remained open to participation for 62 days. Survey dissemination was performed as previously described elsewhere [23,24], using social media and e-mail messaging (examples in Figure S2A). Respondents’ anonymity was ensured by blocking IP identifier recording. Activation of the multiple entry restriction feature was done to prevent participant duplication. Different question formats such as multiple choice, matrix, and open-ended questions were used. SurveyMonkey’s logic feature was added to specific questions to better guide participants and reduce the participation burden [23,24]. Descriptive statistics were performed using Survey Monkey and Microsoft Excel data analysis tools. Graphs were created with GraphPad Prism (version 9, San Diego, CA, USA).

### 2.2. Qualitative Study

The CDG therapeutic R&D tools prioritized through the e-survey determined the think tank discussion topics and were held at the 4th World Conference on CDG for Families and Professionals (Figure 1). A total of 4 think tank discussions were held: (1) CDG biobanks and biomarkers; (2) CDG disease models; (3) CDG registries and NHS; and (4) CDG CTs (Figure 1). All participants who registered for the conference were informed of the think tank sessions in the registration form. Detailed information and instructions were provided to all participants via the conference agenda, booklet, and follow-up emails. During the conference, including in the opening session and immediately before every think tank took place, clear instructions were given by the organizing committee (Figure S2B). Participants were free to withdraw from the discussion at any time.

Nine to ten discussion groups were formed per think tank, entailing 37 discussion groups. The discussion groups involved a total of 90 participants (8–10 per discussion group) and lasted between 15 and 20 min each. Each discussion group had a designated moderator and observers, who respectively led the discussions and annotated the main challenges and solutions identified for each therapeutic research tool. Random assignment of participants to a working group in each think tank was performed using Random Team Generator. It ensured a balanced distribution concerning participants’ background, CDG and therapy R&D experiences, and perspectives. All think tanks were conducted in English and recorded. Transcription was performed by a freelance professional and checked for accuracy by two independent researchers. Content analysis was done as described elsewhere [25]. Briefly, NVivo software (1.3 version, QSR International, https://www.qsrinternational.com/company/about-us) was used to perform an interpretational analysis carried out by two independent researchers (inductive analysis). Open, axial, and selective coding, and the constant-comparison method facilitated the emergence of inductive categories and core themes [26]. First, the data were broken down into tentative categories (open coding). Subsequently, themes emerged through axial coding, i.e., through the identification of connections between the categories. Finally, selective coding, coupled with the constant comparison method, was performed to select core themes. The core themes that emerged were: data management (including data collection, analysis, quality, sharing, and accessibility); sample management (encompassing sample/animal collection, analysis, storage, cataloguing, use, development, sharing, and accessibility); and patient management (namely engagement, recruitment, and participation/accessibility). To facilitate analysis and comparison among the different therapy R&D tools, the challenges and solutions associated with these core themes were grouped into the following categories: (a) administrative, ethical, and regulatory, (b) communication, information-sharing, collaboration, and standardization, (c) financial, and (d) technical, equipment, and biological (Figure 1). Disagreements were settled through discussion and, when necessary, confronted with the respective meeting minutes.

For confidentiality purposes, participant statements were identified by their role in the CDG community (family member, HCP, researcher, industry representative, and multiple roles for those participants who combined various of the previously mentioned roles). Identified challenges and solutions are illustrated by direct quotes drawn from the think tanks. The most representative quotes have been included along the text, and additional illustrative examples have been organized in tool-specific tables (Tables S2–S7).

## 3. Results

### 3.1. A Community-Led Mixed Methods Approach Captures the CDG Community Research Priorities, Perspectives and Experiences

The workflow established for this study encompassed a community-led mixed-methods approach, which succeeded in ensuring representative multi-stakeholder participation in a rare disease community (Figure 1). The quantitative study drew on an e-survey produced and distributed online to identify research priorities focused on CDG therapy R&D tools. Importantly, the data collected in this e-survey also guided and informed the agenda of the 4th World Conference on CDG for Families and Professionals (themed “The CDG road: from diagnosis to therapies”) held in Lisbon, Portugal, in 2019. It effectively converted the conference into a participatory event, tailored to the needs of the participants as well as its potentiated role as a research platform (Figure 1). Accordingly, the qualitative study was planned and carried out during the conference. It comprised four think tank discussions, which enabled the open and detailed debate of the challenges and solutions associated with the prioritized therapy research tools (Figure 1). E-survey and think tank participant demographic data are presented in Table S1. The following sections describe the main findings generated by this community-led exercise, supporting the capability of this approach to deal with the international dispersion of the CDG community, the need to gather insights from a diverse population (professional and family stakeholders) and the complexity of the research topic at hand.

### 3.2. Six Therapy Research Tools Are Priorities for the CDG Community

**Figure 2:**
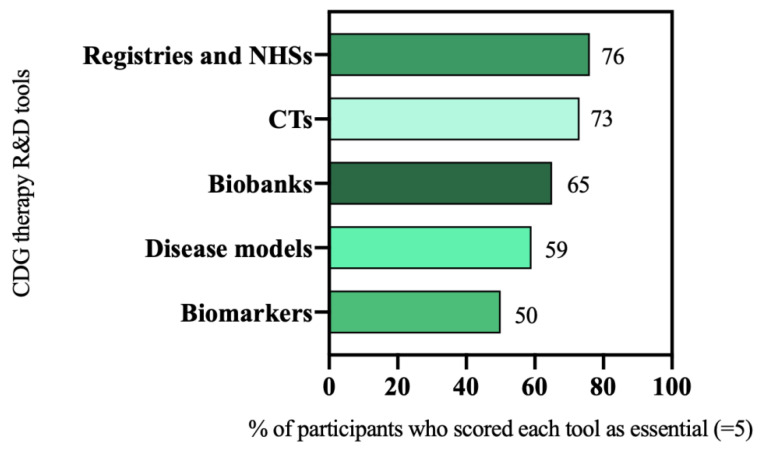
CDG therapeutic R&D tools prioritization.

In the e-survey, participants were asked to rate the relevance of several therapy research tools on a scale of 1 (“I don’t think they are important”) to 5 (“I think they are essential”). Prioritized tools were those scored as being essential (scored 5) for CDG therapeutic R&D by most of the participants (≥50%). The analysis of the e-survey results led to the selection of six R&D tools as priorities, namely: patient registries and NHS (76%, 73 out of 96 participants), and CTs (73%, 94 out of 128 participants), biobanks (65%, 44 out of 68 participants), disease models (59%, 75 out of 128 participants), and biomarkers (50%, 32 out of 64 participants) (Figure 2).

Despite being scored by participants as essential for therapeutic R&D, the participation in and use and awareness of these tools were low (Figure S1). Biobanks and disease models were reported as being used by CDG professionals in their research projects only in 35% (16 out of 46) and 54% (25 out of 46) of the cases, respectively (Figure S1A). Family members showed low familiarity with the disease models available for CDG research (27%, 22 out of 82) (Figure S1B). Only 35% (16 out of 46) of the professionals and 40% (33 out of 82) of the families contributed to or were aware of any CDG patient registry (Figure S1C). As for NHS, few CDG professionals (24%, 11 out of 46) and family members reported (22%, 11 out of 50) ever being involved (Figure S1D). Lastly, in CTs, similar low participation trends were observed among both family members (24%, 20 out of 82) and professionals (31%, 14 out of 46) (Figure S1E).

### 3.3. Data, Sample, and Patient Management Are the Core Elements of CDG Therapy R&D

**Figure 3:**
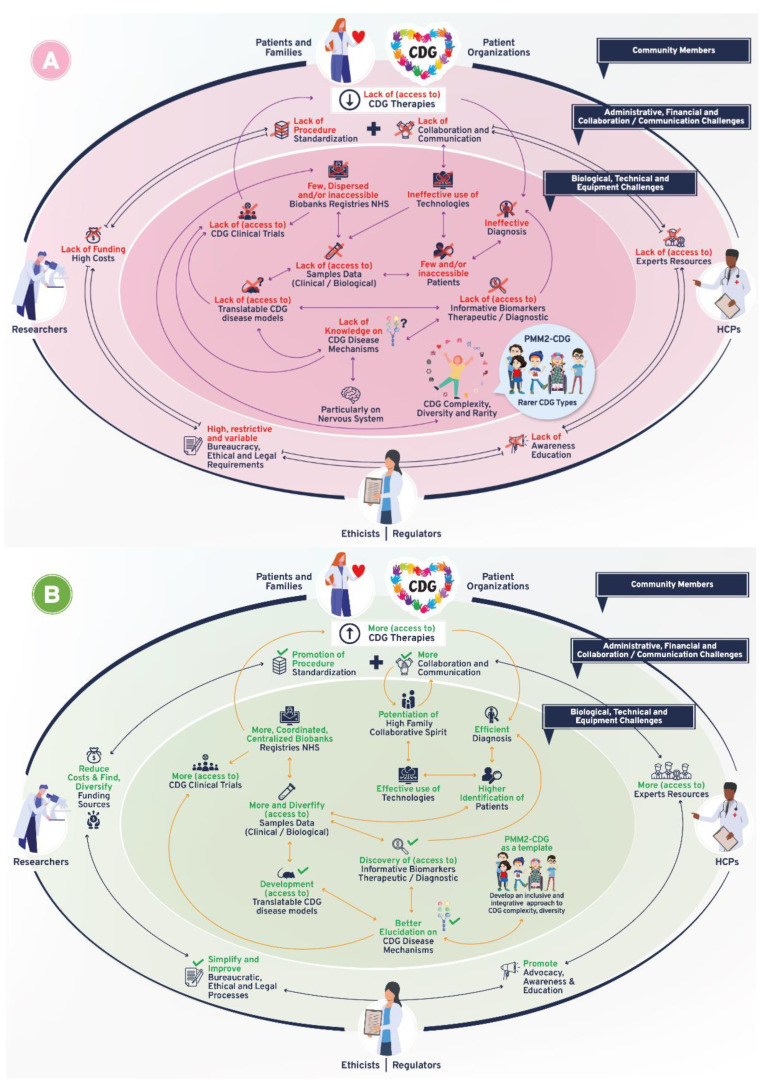

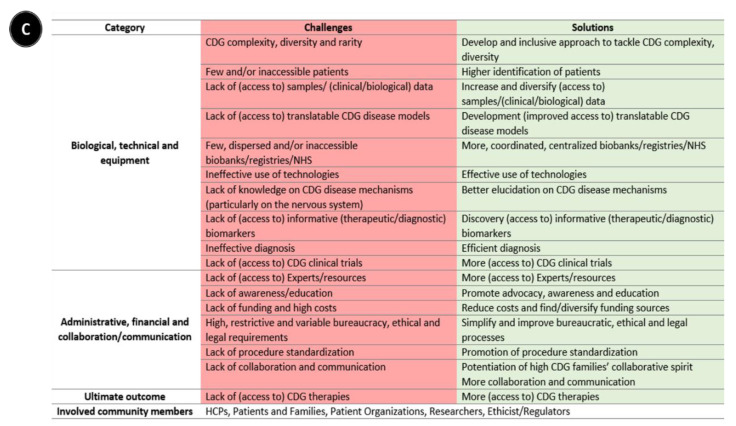
Overview of biomedical therapy-driven research challenges and solutions identified by the CDG community. (**A**) Challenges, (**B**) Solutions, and (**C**) Resume table of panels A and B. On panels A and B, the coloured inside circle shows the biological/technical/equipment challenges or solutions, while the outer circle includes the administrative/regulatory, funds/costs, and communication/collaboration challenges or solutions. The main CDG community members involved in and impacted by these challenges and solutions are depicted in the outer circle. Legend: →—one direction flow; 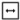
—bidirectional flow; |→—flow interruption or blockage; X—blockage, low levels or quantity, and/or inaccessibility; **√**—availability, increased levels or quantity and accessibility.

Following-up on the CDG community therapy R&D tool prioritization, think tank discussions further revealed data, sample, and patient management as core CDG therapy R&D elements. These elements crisscrossed through the challenges and solutions related to the prioritized tools, which include the following aspects: (a) administrative, regulatory, and ethical; (b) communication, information-sharing, collaboration, and standardization; (c) financial, and (d) technical, equipment, and biological (Figure 1). Figure 3 depicts the global challenges (Figure 3A) and solutions (Figure 3B) identified by the community. The challenges and solutions found are also resumed (Figure 3C) and exhaustively detailed in Supplementary Data with the respective quotes extracted from think tank discussions (Tables S2–S7).

In the next sections, for each prioritized therapy R&D tool, the principal challenges and solutions identified by the CDG Community are presented.

#### 3.3.1. CDG Biobanking (in) Success Is Defined by Sample and Data Sharing and Accessibility 

Biobanks are biosample and data repositories that collect, store, catalogue, and share biological material and the associated data with researchers. These samples and information are used to further understand human diseases and develop therapies [27,28]. The CDG community identified sample and data management as the linchpin of biobanking (Table S2).

##### Challenges of CDG Biobanks

Biobanking sample and data sharing and accessibility were described as affected by various challenges, with administrative, regulatory, and ethical barriers being particularly emphasized (Table S2A). The existence of the restrictive, bureaucratic, country, and/or institution-specific laws and ethical regulations were identified by participants (Table S2A—1.1 and 1.3). These challenges were also linked to sample storage and cataloguing and described to hinder sample traceability while risking sample duplication (Table S2A—1.6). Notably, the research limitations (technical and biological) of not having biosamples accompanied by related clinical and genetic information were highlighted by participants (Table S2A—1.2). Additionally, sample sharing and accessibility associated with communication and information-sharing challenges were often linked to inefficient collaboration, procedure standardization, and unawareness (Table S2A—1.4). These challenges potentiated the creation of collaboration silos within and among research groups/institutions:

*“I feel that one of the main challenges we have is really how scattered the biobanks are (…) there’s actually lots of researcher specific biobanks. (…) in my institution we engage our patients in research and offer skin biopsies for them, and those skin biopsies are generally kept in our institution and so having those accessible to research around the world is a challenge.”*

(multiple roles)

Financial challenges were also pinpointed to constrain sample sharing, storage, and cataloguing (Table S2A—1.5 and 1.7). The high costs related to equipment and to the strict sample storage conditions required to preserve the integrity and quality of the samples were highlighted (Table S2A—1.7 and 1.8).

##### Solutions for CDG Biobanks

The solutions for enabling sample and data sharing and accessibility proposed by the CDG community encompass bureaucratic, ethical, and regulatory processes simplification (Table S2B). Central to these proposals were the engagement of and collaboration among various CDG community members and institutions, such as patient organizations (POs) and the European Reference Networks (ERNs) (Table S2B—2.2). In addition, reinforced family sample and data ownership were also suggested (Table S2B—2.1–2.3). To this end, the role of the informed consent was underlined as a relevant safeguard measure to protect and empower families and patients (Table S2B—2.2).

Better communication and information-sharing through web-based platforms and standardized collaboration between families and professionals were suggested to improve data sharing and accessibility of biological samples, as well as their storage and cataloguing (Table S2B—2.3 and 2.4). Furthermore, participants suggested the creation of a coordinated and/or centralized biosample framework supported by robust collaborative efforts (Table S2B—2.2 and 2.3). Of note, CDG families generally expressed a solid collaborative and proactive attitude towards sample sharing (Table S2B—2.3), which professionals also recognized:

*“We don’t have many problems with parents when we say [that] we have to send blood to someone or [that] we have to send it to another country. They almost always say yes. There’s no problem to collect a little bit more of blood or even skin.”*

(HCP)

#### 3.3.2. CDG Patient Registries Require Efficient Data Management

Patient registries are structured databases that collect longitudinal data—clinical and other—on a population defined by a particular disease. Collected data can help clarify disease presentation and prevalence, playing essential roles in therapy R&D [29]. Accordingly, the CDG community considered data management, encompassing data collection, analysis, quality, sharing and accessibility as key actions to patient registries (Table S3).

##### Challenges of CDG Patient Registries

In CDG patient registries, the identified challenges were reported to differentially impact all aspects of data management (Table S3A). Administrative, regulatory, and ethical constraints were described to transversally affect data management, from data collection to sharing and accessibility (Table S3A—1.1 and 1.5). Noteworthy, legal aspects were underlined, similar to biobanks, as obstacles to data sharing across institutions and countries (Table S3A—1.5). Another challenge pointed out in regard to data sharing was the dispersion of and disconnection among existing CDG registries (Table S3A—1.6).

Participants also mentioned financial challenges mainly connected to data collection, analysis, and quality. These challenges stemmed from the time-consuming and update-dependency of registries, which imposed the need for dedicated, specialized staff responsible for data management (Table S3A—1.1 and 1.3).

Communication problems, mainly related to medical jargon misuse or bias, as well as CDG clinical diversity and complexity, were reported to create several data collection and sharing-related challenges (Table S3A—1.2, 1.4 and 1.7), greatly hindering data definition and interoperability.

The direct involvement of families and patients as data providers in CDG patient registries was highlighted. Families and clinicians identified similar registry participation challenges (Table S3A—1.2 and 1.4). However, the specific communication and comprehension difficulties related to foreign and/or medical language were uniquely reported by families (Table S3A—1.2). Additionally, the possibility of families introducing their data was seen by some professionals as detrimental, causing data quality challenges (Table S3A—1.2).

##### Solutions for CDG Patient Registries

Advanced solutions for CDG patient registries were generally aimed toward the overall goal of promoting effective communication, collaboration, and standardization in all aspects of data management (Table S3B). Shared solutions included (1) making patient registration mandatory (Table S3B—2.1); (2) creating and sharing a protocol for data collection harmonization (Table S3B—2.3); and (3) using existing registries as the foundation to develop a uniform, centralized data repository (Table S3B—2.4). These proposed actions strongly emphasized family and professional collaborations (Table S3B—2.3–2.5 and 2.7). Indeed, a proposal to generate an integrated registry platform shared by families and professionals was received with significant enthusiasm. This common platform was suggested to combine medical and quality of life parameters, allowing the proper integration of the family experience and expertise while guaranteeing the accuracy and quality of the generated data (Table S3B—2.4). Aligned with co-shared platforms and data management centrality, solutions related to communication and information-sharing were emphasized, encompassing the need to promote uniform and explicit language (Table S3B—2.3 and 2.4), ultimately working towards a shared syntax:

*“(…) some sort of common language between patients and professionals”*

(Family member)

Moreover, CDG professionals and families pointed out that keeping communication open and bidirectional was essential, as it effectively motivated data sharing (Table S3B—2.6). Other advanced solutions to facilitate communication and data-sharing entailed using more automatic question formats and technological applications (e.g., multiple choice and apps) (Table S3—2.3 and 2.7).

Besides patient-professional partnerships, POs were referred for their role as information-sharing agents and financial solutions facilitators (Table S3B—2.2 and 2.6).

#### 3.3.3. CDG Biomarker Discovery and Implementation Rely on Robust Sample and Data Collection

Biomarkers are defined as “a characteristic that is objectively measured and evaluated as an indicator of normal biological processes, pathogenic processes, or pharmacological responses to a therapeutic intervention” [30]. Thus, biomarkers play several roles in disease diagnosis, prognosis, and therapy monitoring [31]. The participants reported sample and data management, particularly their collection and analysis, as critical for the discovery and application of CDG biomarkers (Table S4).

##### Challenges of CDG Biomarkers

Challenges impacting biomarker discovery and use in CDG encompassed both data and sample management (Table S4A). Many of these challenges were associated with biological (e.g., CDG clinical complexity, heterogeneity, and rarity) and technical issues (Table S4A—1.2 and 1.5). Notably, important emphasis was put on the difficulties and the need to find CDG neurological biomarkers. These difficulties were predominantly related to the inaccessibility of patient neurological tissues with test invasiveness repeatedly mentioned as a challenge (Table S4A—1.5).

Less referred but still relevant, challenges entailed the regulatory authorities’ resistance to accepting biomarkers, the lack of guidance and information-sharing on existing biomarkers, and the costs of biomarker testing (Table S4—1.1, 1.3 and 1.4).

Altogether, these obstacles contributed to the generalized lack of informative CDG biomarkers for diagnostic, prognostic, and therapeutic purposes:

*“(…) we have been talking about good biomarkers, right? We don’t have them.”*

(researcher)

##### Solutions for CDG Biomarkers

Improving biomarker identification and use requires samples and data. To obtain samples and data, the CDG community urged the need to diversify tissue or sample types as well as to explore new fields, methodologies, and technologies (Table S4B—2.3 and 2.7).

Among other suggested actions to improve biomarker research and their use was the establishment of open communication and information-sharing practices aimed toward collaboration promotion and standardization of data collection (Table S4B—2.2, 2.4–2.6). In addition, the involvement and inclusion of various CDG community stakeholders, namely professionals, families, laboratories, and POs, were reflected in the solutions advanced by the participants (Table S4B—2.1, 2.2 and 2.6). Yet again, the willingness of CDG families to contribute to biomarker research was emphasized (Table S4B—2.6).

#### 3.3.4. CDG Disease Models Require Further Development and Use

Disease models, from simple, cell-based in vitro to complex in vivo organisms, are multi-purpose tools which allow further exploration and elucidation of disease mechanisms, therapeutic target identification, and therapy testing [32]. Think tank participants recognized the relevance of both sample and data management, but CDG disease model (samples and animals) development and use were especially highlighted (Table S5).

##### Challenges of CDG Disease Models

Most identified challenges were related to CDG model development, use, sharing, and accessibility (Table S5A), with the cost of developing and using a model perceived as a very significant bottleneck (Table S5A—1.5). Moreover, disease model development and use were stated to be deeply impacted by ethical and regulatory limitations as well as by several technical, equipment, and strict biological requirements (Table S5A—1.4 and 1.6). Among the latter, the high number of CDG and constellation of presenting clinical signs were pointed out as challenging factors (Table S5A—1.6). Like what was pinpointed to neurological biomarkers, neurological model development was perceived as an added obstacle (Table S5A—1.6). Additionally, lack of knowledge of the underlying disease mechanisms was commented on as a barrier to CDG model use and validation (Table S5A—1.6).

Collectively, these challenges contributed to CDG models failing to faithfully recapitulate the clinical manifestations found in patients (Table S5—1.6):

*“(…) when you’re trying to recapitulate the disease, the animals don’t survive. And when the animals do survive you don’t recapitulate the disease. So, that’s just a fundamental problem that we have”*

(industry representative)

Despite these predominant and fundamental difficulties, data management—including the difficulties in the communication of negative research results and in the use of computational modelling—and model access challenges also emerged (Table S5A—1.1–1.3).

##### Solutions for CDG Disease Models

Participants advocated for solutions towards the successful development, use, and access to CDG translatable models (Table S5B). Interestingly, to achieve this, the creation of an inclusive biological approach to CDG model development and use was put forward. This proposition focused on shared pathomechanisms among CDG, rather than individual models for each disorder:

*“(…) the N-linked, the O-linked, the GPI [glycosylphosphatidylinositol] anchors, perhaps you can learn from one model of one disease, that can apply that to other models within that same pathway”*

(multiple roles)

New methodologies and technologies were also mentioned as solutions to improve model development (Table S5B—2.4 and 2.6). In line with those proposals, opting for simpler disease models (e.g., cells and/or simple organisms such as worms and yeast) was a multi-purpose suggestion. These models were described as economical, easier to work with, and still useful for exploring CDG pathogenesis and even therapy testing (Table S5B—2.5 and 2.6). For instance, induced pluripotent stem cells (iPSCs) were presented as a solution to develop cell or tissue-specific models, including neurological models (Table S5B—2.6).

Given the high costs of model development and use, solutions encompassing the involvement of Pos and public funding were urged (Table S5B—2.3). These measures were found to be important to ensure model sharing and wider accessibility. In addition to these, other models boosting access and sharing solutions were proposed, including the use of sample and animal repositories (Table S5B—2.4). Finally, more solutions with broad impact in CDG disease models entailed suggestions of combining multiple models as data sources optimizing data sharing and standardizing protocols (Table S5B—2.1, 2.2 and 2.7).

#### 3.3.5. Patient and Data Management Are Key Elements for Successful NHS

NHS are longitudinal studies designed to track the course of the disease in the absence of any intervention. Well-planned NHS generate data with versatile applications in therapy development [33]. CDG community members identified patient management—encompassing recruitment, engagement, and participation—and associated data collection, analysis, and sharing as NHS fundamentals (Table S6).

##### Challenges of CDG Natural History Studies

The limited number of the existing worldwide sites is often referred as a challenge for NHS data and patient management (Table S6A). At the root cause of this site scarcity, many challenges were identified, including practical aspects related to costs, administrative, ethical, and regulatory requirements hampering data sharing and/or site selection, and study set-up. In fact, the need to have well-designed, detailed protocols put in place by knowledgeable professionals was recognized as an important obstacle to site expansion (Table S6A—1.1 and 1.2).

In turn, the low number of CDG NHS sites was found to negatively impact patient participation. The underlying advanced reasons were the complicated logistical arrangements and associated significant financial burden of travelling with a medically complex person (Table S6A—1.7 and 1.9). CDG multisystem features and genetic heterogeneity were also identified as challenges affecting patient participation (Table S6A—1.8 and 1.9). Indeed, CDG families described participant burden associated with the multitude of NHS laboratory and imaging investigations as very challenging (Table S6A—1.9).

Communication, information-sharing, and collaboration/standardization challenges were also pinned down as data and patient sharing and participation barriers. Besides inefficient communication between NHS organizers, clinicians, and families, uncooperative professionals and, at times, even possessive attitudes were recognized (Table S6A—1.2, 1.5 and 1.8).

##### Solutions for CDG Natural History Studies

NHS solutions entailed innovative and integrative ways of improving all areas of data and patient management (Table S6B). Accordingly, NHS site expansion was heavily suggested. The options included resorting to international collaborative efforts to create new physical sites, using telemedicine and technological applications to improve data management and patient participation (Table S6B—2.1 and 2.3).

Extending NHS to more CDG was also proposed with a recommendation for retrospective studies, i.e., NHS based on past medical records and test results (Table S6B—2.1). For retrospective NHS, the use of new methodologies, such as artificial intelligence (AI), were a recognized solution to effectively perform these studies (Table S6B—2.1). Moreover, well-designed registries, namely linked to clinical management guidelines, were identified as fundamental resources to simultaneously guide and harmonize NHS data collection (Table S6B—2.1).

Improving data sharing by adopting measures that make communication bidirectional, acknowledged or rewarded collaboration was also suggested (Table S6B—2.2). In that sense, POs, social media, and other virtual channels were identified again as relevant NHS information-sharing and participant engagement catalyzers (Table S6B—2.3). A vital participation booster already documented in other research tools and detected concerning NHS was the altruistic and cooperative spirit of CDG families (Table S6B—2.3).

#### 3.3.6. Patients and Data Realities Challenge CDG CTs

CTs are studies that evaluate the efficacy of an intervention (e.g., a medicine) in the health outcome of a patient population. CTs are finely regulated process-driven ventures. They constitute the last phase of therapy R&D that precedes marketing approval and pharmacovigilance monitoring. The CDG community described patient and data management as the limiting and potentiating CT factors (Table S7).

##### Challenges of CDG Clinical Trials

CDG CT challenges overlapped chiefly with the ones identified for NHS underlying dependency on data management and patient participation (Table S7A). CTs and NHS shared data management challenges associated with study set-up. These challenges included administrative, regulatory, and ethical requirements, financial demands, in addition to equipment, technical and other biological constraints (Table S7A—1.1–1.3). Other data sharing and accessibility difficulties were also named, including result communication bias linked to the used channels (e.g., PubMed) and the lack of tradition and expertise in sharing negative results with patients and families (Table S7A—1.4). Several challenges impacting patient engagement, recruitment, and participation, such as the low number of CTs, sites and medicines being tested, were deeply emphasized (Table S7A—1.5). Costs and the lack of access to information, infrastructures, and experts were also described as additional obstacles to patient engagement and participation (Table S7A—1.5–1.7). Even though having a less common CDG was stated to be a participation obstacle (Table S7A—1.7), just like in NHS, in CTs PMM2-CDG dominance alongside the marked sense that other CDG felt overlooked were clearly transmitted by the participants:

*“(…) all of my patients that don’t have PMM2-CDG think PMM2-CDG is getting way too much attention (…) All CTs are for PMM2-CDG, but that doesn’t actually serve us well, because if we want to say that we’re a CDG group, then we need to be inclusive”*

(HCP)

Interestingly, the CDG community adamantly anticipated a new challenge arising from PMM2-CDG therapy R&D domination: the possible (and potentially harmful) PMM2-CDG patient population fragmentation resulting from the multiple therapies under trial (Table S7A—1.7).

##### Solutions for CDG Clinical Trials

As expected, CDG CT solutions also greatly overlayed with NHS-proposed measures (Table S7B). Participants proposed transversal solutions to data and patient management-related challenges, including better communication, collaboration, and early and inclusive stakeholder involvement. Regarding communication and collaboration-related solutions, open and standardized communication through online channels (e.g., protocol and result sharing), and reinforcing the informed consent as a CDG patient/family protection and communication tool were also proposed (Table S7—2.1 and 2.5). These solutions implicated various stakeholders, namely regulators, clinicians, Pos, and families (Table S7B—2.1, 2.2, 2.4 and 2.5). Although the powerful and differentiated role of CDG families had already been noted concerning other CDG therapeutic R&D tools, it was especially underlined in CTs. Besides expressing a selfless and pro-research attitude towards CTs, CDG patients and families were identified as essential data collection and study recruitment partners, and as stakeholders able to bypass impeditive logistical hurdles (Table S7B—2.1, 2.4 and 2.5). A generalized idea that emerged was the need for more CT sites, more therapeutic avenues, and ultimately, more therapeutic R&D—including more CDG and patients. For CT site expansion, telemedicine was a mentioned solution (Table S7B—2.3). In terms of exploring therapeutic options, drug repurposing and sugar supplementation strategies were identified as faster, cost-effective, and helpful symptom-management strategies for CDG CTs (Table S7B—2.2 and 2.3). Lastly, broadening the CT spectrum to include more CDG either by grouping CDG together and/or by using PMM2-CDG as a template was an advocated solution:

*“(…) grouping small groups of patients together (…) with a similar pathogenesis to PMM2”*

(industry representative)

### 3.4. Diagnosis and Therapies Are the Bidirectional Triggers of CDG Therapy Research Tool Interdependence

**Figure 4:**
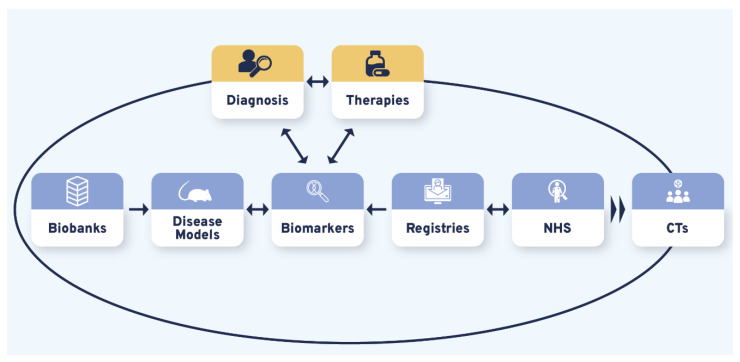
The CDG research tools interdependency. Variable and multiple interconnections were established between the CDG therapy R&D tools with all influencing CTs. Diagnosis and possible therapies were identified as essential triggers for the CDG therapy R&D flux, represented by the larger black circle. The smaller blue, orange, and yellow circles depict the uncovered reciprocal associations between different research tools. Legend: CTs—Clinical trials; NHS—Natural history studies.

Therapy R&D is often represented as a linear process whose success relies on various and variably interconnected tools. Our results showed that the different therapeutic research tools prioritized by the CDG community produced CDG therapy R&D interdependency circles. Although bidirectional connections were uncovered between and among the studied therapy research tools, the CDG community firmly underlined this about CTs by recognizing they were strongly reliant on the other (upstream) therapy research tools (Figure 4, Table S8). Notably, the identified triggers of these interrelational circles were diagnosis and therapies (Figure 4):

*“(…) it’s a bit of a vicious circle, (…) we don’t have enough biomarkers to accurately diagnose, but without an accurate diagnosis families can’t get into the system, to be enrolled in the biobanks, to permit the development of biomarkers (…) it’s shortening that “diagnostic odyssey” (…) so that I as a patient can get to see you as an expert quickly, and you can take my samples and put them into the biobank, to do the research to find the biomarkers (…)”*

(multiple roles)

Moreover, participants observed the existence of a bilateral relationship between CDG diagnosis and treatment:

*“(…) we don’t even have [CDG] newborn screening, because most clinicians think it’s not treatable (…)”*

(HCP)

The timing of diagnosis was also discerned as a CDG therapy R&D effectiveness modelling component:

*“(…) a lot of older patients (…) can benefit from [management therapies] whereas a cure should be preferably implemented by early diagnosis”*

(family member)

These results suggest that working towards improving one of the therapy R&D and/or diagnostic tools will benefit other tools. Ultimately, all the advances will be translated into CT acceleration and therapy development.

## 4. Discussion

Therapy R&D entails multiple tools and the involvement of various stakeholders with different biological, medical, and technical expertise. However, other factors affect drug discovery and testing including ethical, legislation, and funding issues, some of which are particularly difficult to address in the field of rare diseases [17,18]. Our study established a unique, replicable community-led research approach that involved both patients’ families and professionals able to collect and integrate the perspectives, priorities, and experiences of all stakeholders regarding therapy-driven biomedical and clinical research. This pioneering approach produced a detailed characterization of the CDG therapy R&D landscape, establishing several points of contact with other rare disease communities. It also unveiled that CDG therapy R&D success sits on six prioritized research tools, which is limited by several challenges that constrain the appropriate management of data, samples, and patients. The CDG community proposed various solutions that can be deployed to address those challenges, promote people-centric therapy R&D, and inspire other rare disease communities to devise actions to tackle similar therapy research-related problems.

CDG clinical diversity, biological complexity, and rarity were described to deeply affect most of the therapeutic R&D tools included in this study. It was evidenced, for example, by concerns with the currently incomplete understanding of CDG pathophysiology. These challenges were particularly expressed regarding neurological involvement and further reinforced by the lack of informative biomarkers, difficulties in establishing meaningful clinical endpoints, and valid disease models. The recentness and complexity of the glycobiology and CDG fields were advanced as underlying causes of several technical difficulties, including those inherent to the study of glycans and the nervous system [1]. Nevertheless, according to the CDG community and published literature, new technologies and methodologies can be relevant problem-solving approaches in these settings [2,8,34,35]. Suggestions included the gene editing CRISPR-Cas9 technology, computer modelling and data analysis using AI, as well as the application of (multi-)omics (e.g., glycomics and metabolomics) approaches in the development and identification of more reliable disease models and biomarkers.

Moreover, technological applications, particularly online tools and platforms, are becoming consolidated communication and information-sharing channels used for patient engagement and recruitment in therapeutic research [23,24,36]. CDG community members approved these web-based communication practices to boost patient participation in therapy research. Additionally, the advent of eHealth (closely related to AI applications and encompassing electronic medical records) has been revolutionizing this new borderless field of medicine [37]. The CDG community proposed telemedicine as a solution to improve data collection and promote (both NHS and CT) site expansion. Telemedicine development is undeniable with some ongoing international efforts, with ERN-driven efforts deserving a particular highlight [38]. The multiple advances and uses of telemedicine are especially relevant as rare disease patients are clearly supportive of data sharing to foster research and improve healthcare [39]. Following what is reported by Courbier and colleagues regarding rare disease patients and families, our results showed that CDG families and professionals defend strong family and patient control over their data and samples [39]. The CDG community believed that establishing clear family ownership would solve many sample- and data-sharing challenges. In our study, the informed consent was put forward as a powerful tool to achieve improved family/patient control. Additionally, the consent containing an option for result disclosure was suggested as an effective option to facilitate patient and family access to updated information about therapeutic research and development.

Biobanks and registries emerged as strongly administrative-bound tools concerned with sample and data management. Solutions put forward by the participants were based on the standardization of data and sample management procedures following FAIR (Findability, Accessibility, Interoperability, and Reusability) principles [40]. Successful examples of sample and data management harmonization for efficient therapy development and approval have been achieved by biobanking and registry-linked initiatives [27,28,29,40]. Hence, there are models from which the CDG community could draw information and inspiration to implement these measures effectively.

Think tank participants also contributed with innovative suggestions, such as using CDG clinical management guidelines to define registry data collection and guide NHS set-up and development. Besides promoting data standardization, this proposal blurs the line between clinical practice and research. It also highlights the interdependence between the different therapy research tools, i.e., how the development or implementation of one tool is influenced by or relies on other(s). Hence, improving a research tool will positively affect the other(s). Concomitantly to this interrelationship, our data unveiled two bilateral triggers—diagnosis and therapies which also regulate each other. Participants stated that the label “untreatable disease” can by itself delay diagnosis while, at the same time, the existence of treatment is a decisive criterion for a particular disease to be included in newborn screening programs [41]. Ultimately, this means that ameliorating diagnosis across diseases perceived as incurable, such as CDG, can boost therapy development and vice-versa.

Similarly to 95% of all rare diseases, CDG have no approved therapies yet [42]. Still, the community identified disparities in access to several research tools, including therapies under study. Drug repurposing is a strategy gaining momentum in rare diseases, including CDG [8,10,11]. Participants expressed positive views on drug repurposing since it was perceived to reduce therapy R&D costs and time.

A common underlying thread was present across many of the therapy R&D solutions proposed by the CDG community: better collaboration. The implementation of multidisciplinary and international collaborative networks has been a long-defended and increasingly adopted solution in rare disease research and care [42,43]. In CDG, there are two main initiatives: the Frontiers in CDG Consortium (FCDGC, [44]) and the international patient-led CDG & Allies—Professionals and Patient Associations International Network (CDG&Allies-PPAIN, [45]). Besides potentiating and standardizing therapeutic research, multistakeholder partnerships have shown potential for more effective information-sharing and funding initiatives [42,46,47]. POs are gradually being more considered research partners, especially in rare diseases, as they have been playing increasingly important roles in therapy R&D [27,33]. In CDG, POs have been involved in NHS collection via questionnaires and patient registry implementation [23,24,48]. Accordingly, patient registries emerged as the research tool where the direct CDG patient/family involvement is most well-established.

Moreover, medicine regulatory authorities’ increasing use and valuing of quality of life measures by medicine regulatory authorities have resulted in a paradigm shift towards the active involvement of families and patients as partners [49]. Our data reasserts a long-lasting pro-research and collaborative spirit among CDG families [25], a feature also documented among other rare disease communities. This attitude can act as a vital leveraging factor in the adoption and efficient implementation of various proposed solutions [33,39].

### 4.1. Study Limitations

- E-surveys were only made available in English, which might have influenced participation. However, in previous e-survey-based studies distributed among the CDG community, the English version was the most represented [23,24];
- Think tanks were also exclusively conducted in English. They may have led to the underrepresentation of participants’ views with limited English proficiency while favoring native speakers expressing their opinion and sharing their insights. In order to tackle this potential limitation, command of English and other languages (according to the nationality reported in the registration form) proficiency was a weighed factor when assigning participants to the think tank groups. Additionally, during the think tank discussions, participants were stimulated to express themselves in their mother tongue when necessary, benefiting from translation into English by another discussion group member;
- Our study may have been subject to selection bias, particularly regarding recruitment for the think tanks, which occurred in connection with the 4^th^ World Conference on CDG. Conference participants tend to be more engaged, academically educated, and/or financially empowered participants when compared to the overall CDG community [25].

### 4.2. Study Strengths

- The development and implementation of a sustained participatory, community-led study including lay and professional stakeholders from diverse backgrounds and nationalities enabled the creation of a comprehensive map of the current CDG therapeutic research landscape and the offering of orientation for future therapy R&D. Importantly, the reproducibility of this approach offers the possibility of its adaptation by other disease communities and/or for other biological questions;
- The adoption of mixed research methods enriched the completeness and depth of the data. At the same time, it made data collection a stepwise process, guiding data analysis and strengthening study conclusions;
- The e-survey methodology used to gather quantitative data, including survey development and dissemination, has been optimized by our research team in previous works [23,24]. Regarding the qualitative study, the key strength of the think tank methodology is based on the possibility of participants meeting face-to-face in a safe environment which stimulates sharing, reflection, and co-learning. The previous identification of the World Conference on CDG for Families and Professionals as a collaborative platform aided and added another level of innovation to the study design [25]. Framing the study around the World Conference on CDG also allowed the community to co-develop the conference agenda according to their needs and preferences. These multiple study outcomes underscore the adaptability and positive impact of people-centricity in research.

## 5. Conclusions

Our community-led, participatory methodology confirmed that CDG therapeutic biomedical and clinical research is faced with many—at times common and interlinked—challenges and amenable to solutions found across other disease communities. In turn, many of these findings will resonate with and benefit other disease fields and communities, particularly other rare and/or complex disorders. Additionally, having a well-developed advocacy movement is vital for successfully implementing the proposed methodology. Two examples of rare communities where this condition is met are the Duchenne muscular dystrophy and the Cystic fibrosis communities.

In summary, this study identified collaboration and standardization across all areas of data, sample, and patient management as common proposals to effectively tackle and solve the financial, technical, administrative but also biological challenges of CDG therapy R&D. Noteworthy, the participants suggested using CDG clinical and genetic diversity as an advantage to therapeutic R&D by building an inclusive approach that leaves no CDG patient behind.

To conclude, we believe this methodological approach promotes patient-centered care, i.e., brings the patient community care needs to the table, promotes co-working and co-decision-making between professionals and families, and empowers CDG families and patients to take action. In addition, the information collected in this study is precious for improving CDG therapeutic research, informing long-term CDG care plans and strategies, and guiding future health care decisions.

## Data Availability

All data generated or analysed during this study are included in this published article (and its supplementary information files).

## Supplementary Materials

The following supporting information can be downloaded at: https://www.mdpi.com/article/10.3390/ijerph19116829/s1, The supplementary material consists of figures and tables supporting the results presented.
