# Supplementary material for: A Community-Led Approach as a Guide to Overcome Challenges for Therapy Research in Congenital Disorders of Glycosylation
Source: Int J Environ Res Public Health. 2022 Jun 2;19(11):6829. doi: 10.3390/ijerph19116829 (PMC9180837; doi:10.3390/ijerph19116829)
Supplement: Supplementary file 1 [file ijerph-19-06829-s001.zip › ijerph-1652590-supplementary.pdf]

Table S1. E-surveys and think tank participant demographics.

Participant characteristics	E-surveys (N=128)	Think Tank discussions (N=149)
Role in the CDG Community		
Family member	82	72
HCP[a]	21	28
Researcher (clinical or non-clinical)	25	25
Industry representative	NA	11
Multiple roles[b]	NA	13
Area of residence (continent)		
Europe	76	111
America	37	28
Other	15	10

Legend: [a] – HCPs include clinicians, nurses, and laboratory technicians with a primary role in routine clinical practice; b – This category was created in the think tanks and encompasses participants who combined more than one of the above-mentioned roles (e.g., a clinician who is also a clinical researcher); HCP – Healthcare professional; NA – Not available/applicable

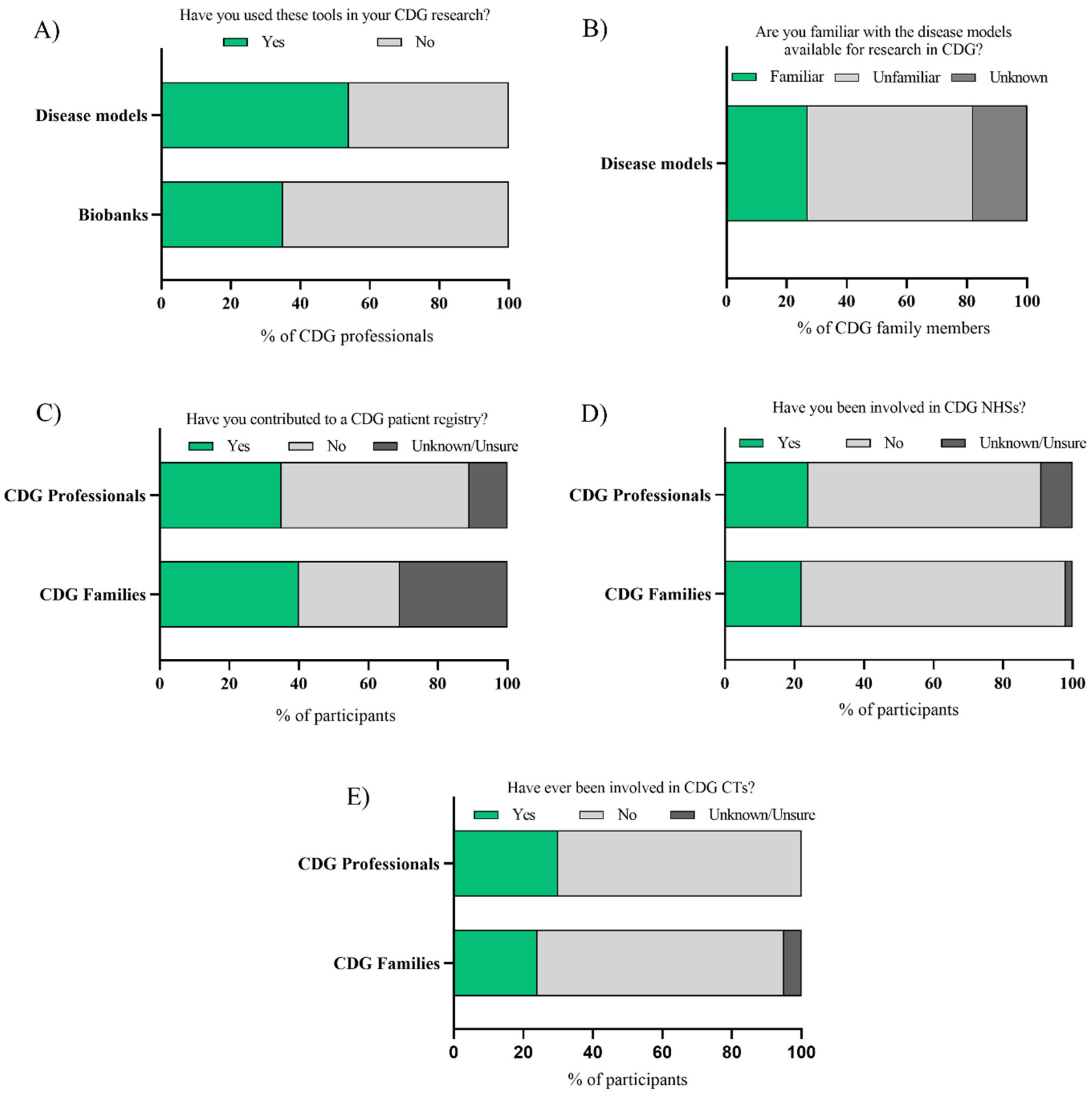

Figure S1. CDG therapeutic R&D use, awareness, and participation among CDG family members and professionals. A) Use of biobanks and disease models by professionals in their CDG research; B) CDG families' familiarity with CDG disease models used in research; CDG families and professionals participation C) in CDG patient registries, D) in natural history studies (NHS) and in E) clinical trials (CTs).

Table S2. Challenges and solutions of CDG biobanks

Biobanks Theme	Sub-theme	A. CHALLENGES	B. SOLUTIONS
**Data management**	Data sharing and accessibility	**1.1. Administrative/regulatory/ethical**<br>" *(...)the new European law, the GDPR [General Data Protection Rules] (...) makes it almost impossible to get this [sharing data alongside the sample] in place (...) moving personal information is almost impossible in Europe today*" (industry representative)<br><br>**1.2. Technical, equipment and biological**<br>*"I think it's very important because if you just send blood or fibroblasts without information about the diagnosis, the severity, the age, then it's not useful"* (multiple roles)	**2.1. Administrative/regulatory/ethical**<br>"*But I think if the need [of sharing clinical information] is coming from the patient, then maybe the patient is OK with sharing*" (family member)
**Sample management**	Sample/animal sharing and accessibility	**1.3. Administrative/regulatory/ethical**<br>*"(…) sometimes the researcher needs samples, and as a family we want to do it, but we can't, because (…), we have a lot of bureaucratic problems to do it"* (family member)<br><br>*"(…) but there is different legislation for biobanks in South Africa compared to Europe, compared to America, and that makes it also a little bit difficult in sharing data and samples within the biobanks"* (HCP)<br><br>**1.4. Communication, information-sharing and collaboration/ standardization**<br>*"I must admit as a doctor that we do not make any sampling for the biobank when we have a CDG patient, because we make all the analysis by ourselves"* (HCP)<br><br>*"(…) as soon as a family is diagnosed submitting a biosample is not part of like the checklist of things that is just customary or seen as obligatory"* (researcher)<br><br>*"I don't know if there is any biobank in France, I don't know how to give samples to them"* (family member)	**2.2. Administrative/regulatory/ethical**<br>"*Everything is in the consent that you sign with the hospital to share the samples with the biobanks or to give the samples to the researchers for fundamental research (…) [It is] your right to be informed of the results of the project where you have signed your consent"* (multiple roles)<br><br>" *(...) a link between the doctor, patient association and then a research consent with the samples to the patient association, in a way that [the informed consent] is broader*" (researcher)<br><br>"*(...) the ERNs [European Reference Networks] can make this possible, because they have special European law attached to their functioning, so they are already in a network (...) all these centers are in there, so I think that they're happy to share their samples (...)*" (Multiple roles)<br><br>"*We should have a reference center in each country to centralize the samples*" (HCP)<br><br>**2.3. Communication, information-sharing and collaboration/ standardization**<br>*"We need the addresses of every biobank (…) on the CDG website"* (family member)

Biobanks Theme	Sub-theme	A. CHALLENGES	B. SOLUTIONS
		**1.5. Financial**<br>*"(…) if I want to send cells from Australia to Leuven, it would cost me 3 thousand dollars (…)"* (multiple roles)	*"Maybe creating a platform that includes the patients, and the physicians and the researchers. So, each patient diagnosed with CDG can just submit his sample easily, with a proofed consent, so even he can get access to his sample progress, any updates about his sample from the researcher (…)"* (HCP)<br><br>*"(...) If the families come to a consensus and discuss with their hospital treating physician how to be able to ship the samples that are being stored in freezers all over the world to a good, centralized place (...)"* (multiple roles)
	Sample storage and cataloguing	**1.6. Administrative/regulatory/ethical**<br>*"So the samples have to be blinded, they have to be accessible with a fair amount of information (…) you need to keep those records for an indefinite period of time, you need to be able to know how many specimens you have (…), you have to have a record of where you've spent those specimens"* (industry representative)<br><br>*"(...) you can provide cells anonymized with a diagnosis and maybe symptoms, but it's very hard to connect it via an identifiable tag. And if you can't do that, it's very hard to keep patients separated (…) You have duplicates, and triplicates"* (industry representative)<br><br>**1.7. Financial**<br>*"making sure that those samples are stored properly, so if somebody gets an idea later, if they want to test it, those samples could be available. I think that's going to be big, so cost rate"*(family member)<br><br>**1.8. Technical, equipment and biological**<br>*"they had to be stored at refrigerated conditions and never deviate from that temperature for 5 or 10 years (…) because the samples are spoiled"* (industry representative)	**2.4. Communication, information-sharing and collaboration/ standardization**<br>*"(…) make sure that the sample that gets to a biobank is well described. (...) some co-working [families] with the professionals to make sure that the sample gets into the right biobank part"* (HCP)

Table S3. Challenges and solutions of CDG patient registries

Registries Theme	Sub-theme	A. CHALLENGES	B. SOLUTIONS
**Data Management**	Data collection, analysis and quality	**1.1. Administrative/regulatory/ethical**<br>*"(…) the main problem with the registries is [that] they give a lot of work, someone has to introduce the data, confirm the data (...) and update the data (…)"* (HCP)<br><br>*"(…) you have to go through (…) tons of paperwork and the [ethical] committee they don't understand what you're talking about at all (…) they don't understand even what CDG is"* (HCP)<br><br>**1.2. Communication, information-sharing and collaboration/ standardization**<br>*"I think also for professionals, because sometimes you use different terms for the same sort of symptom (...)"* (HCP)<br><br>*"I don't know how to write in English (…)"* (family member)<br><br>*"It's also difficult for us (…) parents, to fill out such forms, because sometimes we mix up some medical terms. Sometimes I don't know what to put in, because the way the question is put is difficult to understand…"* (family member)<br><br>*"It may not be that useful if patients start to also introduce clinical data, because things which are not correctly entered into a system could really mess up the study*" (HCP)	**2.1. Administrative/regulatory/ethical**<br>"*(...) the obligation of declaring all the patients. So, you can direct these patients to one single center or not, so make a very exhaustive registry of every country*" (multiple roles)<br><br>**2.2. Financial**<br>*"(…) to take advantage from some call from the European Union about a creation of a registry for CDG at the European level at least"* (multiple roles)<br><br>*"Maybe it is possible to collaborate, USA – Europe, to create the registry, but I believe that the families, the family associations have the strength to promote this type of projects, to have a financial support"* (researcher)<br><br>**2.3. Communication, information-sharing and collaboration/ standardization**<br>"(…) *protocol to be sure that in each country we are going to ask the same things to all the parents to have a good registry and to make good comparisons between different things: the behavior also, the effect of the environment*" (family member)<br><br>"*(…) just from talking to other families, I think things arise, for example, overeating is something that's always been a major problem for my child, and it's only really now beginning to be recognized*" (family member)

Registries Theme	Sub-theme	A. CHALLENGES	B. SOLUTIONS
		**1.3. Financial**<br>*"The main problem is the financial support, to pay the personnel to do the registry and also to do the database"* (researcher)<br><br>*"(...) it is money, right? Because otherwise you could hire somebody to do it [enter the data], because you have all the information"* (industry representative)<br><br>**1.4. Technical, equipment and biological**<br>*"(…) how to put into the registry the things that are not easy to measure (…) what about behavior, what about neurological signs, what about feeding difficulties, not just dysphasia but like taste restriction (…)"* (multiple roles)<br><br>*"(…) difficult to make the same questions for different types, because PMM2[-CDG] is really different from ALG1[-CDG]"* (multiple roles)	"*(…) do it together [family] with the clinician, or to have more clear instructions, examples, multiple choice, (...) to use more lay language for the questionnaires*" (industry representative)<br><br>**2.4. Technical, equipment and biological**<br>*"One of the tools to solve some of these huge problems [data collection and registry implementation] may be to achieve information from a variable dataset that each clinician already has (…) [it] could be a first step in order to create this platform"* (multiple roles)<br><br>"*(…) if we can go in a direction that somehow we unify them [clinical symptoms] and we say that 312, that's a number for this sort of symptoms*" (HCP)<br>"*(...) to make this registry we need to mix your questions [Patient Reported Outcome Measures] and the medical questions*" (multiple roles)<br><br>"*One of the most important things, besides the collaboration of the families, is to have those registries validated by the clinicians*" (HCP)
	Data sharing and accessibility	**1.5. Administrative/regulatory/ethical**<br>*"(…) there's probably still some concerns around data going to other countries that don't necessarily have the same levels of privacy(…)"* (family member)<br><br>*"Maybe the problem (…) is who is the owner of the data, who is going to make the publications (…) And then the data protection too, (…) because the power of this data is similar to the power of the biobank. I mean, it is really important data to protect"* (multiple roles)<br><br>*"(…) for legal reasons, you maybe cannot also share information between hospitals."* (HCP)<br><br>**1.6. Communication, information-sharing and collaboration/ standardization**	**2.5. Administrative/regulatory/ethical**<br>*"When you ask a parent if we would share, we say, yes, share (…) share it all three registries or whatever there are, right? Like, I'd be happy to"* (family member)<br><br>**2.6. Communication, information-sharing and collaboration/ standardization**<br>"*(...) the doctor that receives the diagnosis and hands it to the patient could also say, now you should contact this people [patient support group] (…) I think that would by itself increase the numbers [of registered patients] a lot"* (industry representative)<br><br>*"It's very important that we have feedback on the data that we send (…) that will motivate us to go on"* (HCP)<br><br>"*I think it will be wonderful for the registry because you [the families] would be also able to get back the data, like if you provide your data, you can ask the*

Registries Theme	Sub-theme	A. CHALLENGES	B. SOLUTIONS
		"(…) *there's not a lot of them [CDG registries] but they're already small and fractured (…)*" (multiple roles)<br><br>"*when it comes to MetabERN [registry] (…) I have not been approached, so I could submit (...) 35 patients and you see 5 in there (…)*" (multiple roles)<br><br>"*I didn't know of CDG Connect [CDG family-fed registry] until today. So, having an asset is one thing, creating awareness is another thing*" (multiple roles)<br><br>**1.8. Technical, equipment and biological**<br>"*(...) it is difficult to mix or to join together all the data, because every registry has different questions*" (multiple roles)	*question: how many other patients with the same diagnosis are there (…) from my country*" (multiple roles)<br><br>**2.7. Technical, equipment and biological**<br>"(…) *to have a platform for both [families and professionals], because I know from my patient group, there are some families, so very motivated to do it [participate in the registry]*" (family member)<br><br>"*Making use of technology, I think you would rather have an app on your phone (…). You will be more prone in completing (…) the information*" (HCP)

Table S4. Challenges and solutions of CDG biomarkers

Biomarkers Theme	Sub-theme	A. CHALLENGES	B. SOLUTIONS
**Data Management**	Data collection, analysis and quality	**1.1. Administrative/regulatory/ethical**<br>*"(…) one of the biggest challenges with biomarkers is getting FDA [Food and Drug Administration] and regulatory authorities to accept them or acknowledge their value"* (industry representative)<br><br>**1.2. Technical, equipment and biological**<br>*"Technically, we're still not there (…) which is part of why CDG gets forgotten, because that's so challenging to study the sugar components."* (industry representative)<br><br>*"the pathway that affects transferrin, really only encompasses about half of the CDG (…). And the big challenge with transferrin as a biomarker is that in many of the CDG at some point it will spontaneously normalize (…)"* (multiple roles)<br><br>"*(…) there's so many systems involved in CDG and there's such an array of phenotypes. What would be a good biomarker? A biomarker for one person might not be a biomarker for another person with the same CDG*" (family member)	**2.1. Administrative/regulatory/ethical**<br>*"I would hope that advocacy groups, other researchers and the community as a whole can do for CDG is to really increase the pressure and increase the advocacy for regulatory authorities to accept them [biomarkers]"* (industry representative)<br><br>**2.2. Communication, information-sharing and collaboration/ standardization**<br>"(…) *there has to be an agreement among clinicians about what you're measuring regularly. And then there also has to be a negotiation with the [regulatory] agencies*" (industry representative)<br><br>**2.3. Technical, equipment and biological**<br>"*Metabolomics yes could be helpful for biomarkers, but also doing glycomics, glycoproteomics, and those things to see if that might be closer to the disease*" (multiple roles)<br><br>"We *need other biomarkers (...) I think we need more research and some of that comes from collecting routinely tested things"* (multiple roles)
	Data sharing and accessibility	**1.3. Communication, information-sharing and collaboration/ standardization**<br>"(*...) we don't have clear guidance on when and what biomarkers we can measur*e" (HCP)	**2.4. Communication, information-sharing and collaboration/ standardization**<br>*"(...) getting a list of laboratories and biomarkers they found useful, communicate this within the laboratory community, (…) having a laboratory proficiency sharing"* (researcher)<br><br>**2.5. Technical, equipment and biological**<br>"*getting the experts opinion, so creating at least a cocktail of biomarkers, so they can guide the physicians*" (HCP)

Biomarkers Theme	Sub-theme	A. CHALLENGES	B. SOLUTIONS
**Sample management**	Sample collection and analysis	**1.4. Financial**<br>"*His lab does the diagnosis but then we send the samples, who is paying? Probably his lab is taking all the expenses, because there are not proper collaborations or proper agreements*" (researcher)<br><br>**1.5 Technical, equipment and biological**<br>"*(…) the involvement of the nervous system, which is very difficult to study, very precise and very difficult to obtain biomarkers from*" (researcher)<br><br>"*We know what's in urine, and we know what's in blood, we know what's in CSF [cerebrospinal fluid], but the problem is a lot of these disorders do not express themselves in these tissues and getting biomarkers from the other tissues obviously is hard, because you don't have that tissue. You don't do a brain [biopsy] (…) muscle is a problem, I mean it's easy to do a muscle biopsy, but it's still invasive in a way*" (industry representative)<br><br>" (…) *the problem with the size of the cerebellum is that it is hard to measure, you need an MRI [magnetic resonance imaging], to sedate the child, that's a big test*" (multiple roles)<br><br>"*not all labs have the tandem mass spectrometry and the facility to analyze glycoprofiling in plasma, so it is one difficult task to perform, a general screening for biomarkers(*...)" (HCP)	**2.6. Communication, information-sharing and collaboration/ standardization**<br>"*when we explain to the parents what we are going to do [skin biopsy], (...) they [families] say yes (…)*" (HCP)<br><br>"*I think that it [a lumbar puncture] is invasive, but if the marker is clearly identified, and if I know what the objective is (...), I will be OK*" (family member)<br><br>**2.7. Technical, equipment and biological**<br>"*(…) by using metabolomics instead (…) and different body fluids, like saliva or urine (…) That would be also good for the patient that you don't have to take blood all the time*" (multiple roles)

Table S5. Challenges and solutions of CDG disease models

Disease models<br>Theme	Sub-theme	A. CHALLENGES	B. SOLUTIONS
**Data Management**	Data collection, analysis and quality	**1.1. Technical, equipment and biological**<br>"(…) *on the systems biology aspect of AI [Artificial intelligence], I think if we can develop a system where you can do big data analysis (...) with modelling and with computer software it is the data they collect that is important (…) if you get bad data, you get bad results (...)*" (industry representative)	**2.1. Technical, equipment and biological**<br>"*I think it's good if you can have multiple models, different animal models, different cell models, then all the evidence start to give you confidence in your mechanism of action*" (industry representative)
	Data sharing and accessibility	**1.2. Communication, information-sharing and collaboration/ standardization**<br>*"many labs (…) try to (…), develop iPS [induced pluripotent stem] cells (…) it was not successful (…) we know only the positive results"* (researcher)	**2.2. Communication, information-sharing and collaboration/ standardization**<br>"*I think it's really very important to share the different studies [results]*" (family member)
**Sample management**	Sample/animal sharing and accessibility	**1.3. Communication, information-sharing and collaboration/ standardization**<br>*"Actually access, finding people that have the mouse models is very difficult. In Ageos [a public biotech company] they had a PMM2[-CDG] model, but you could only use it if you worked out a deal with them, (…) even finding good access to cell lines has been challenging"* (industry representative)	**2.3. Financial**<br>*"And if the patient groups do it [fund disease models], then they can also own those models and then they can take those models and give them to other researchers and spread them around"* (researcher)<br><br>"*It really takes funding to make the right thing [disease model] and having the funding be public so everyone can get it [disease model]*" (industry representative)<br><br>**2.4. Communication, information-sharing and collaboration/ standardization**<br>"*(...) kind of a company that has [a library of] iPSCs [induce pluripotent stem cells] or the CRISPR-Cas9 [gene editing technology] cells (...) you buy the cells from the library*" (researcher)<br><br>"(…) *if someone has a mouse model that maybe we can share other materials, so not necessarily the whole animal, because we cannot transport that, but we can use some bone marrow, or some spinal fluids or some brains, you can take everything from a mouse and send it somewhere else*" (researcher)

Disease models<br>Theme	Sub-theme	A. CHALLENGES	B. SOLUTIONS
	Sample/animal development and use	**1.4. Administrative/regulatory/ethical**<br>"*(...) the ethical problems with the use of animals nowadays. It's another problem*" (researcher)<br><br>"(…) *time to maintain the colonies and investigate all of their phenotypes and make sure that things don't change over time, it's just a lot of work and a lot of time. Then when the regulators get involved and they want to have control of everything*" (industry representative)<br><br>**1.5. Financial**<br>"*(…) you have to develop this model, it's few years to develop, and (…) you've collected animals, not have only one, but multiple (…) the feeding, and for the staying of the animals, it's very expensive*" (HCP)<br><br>"*Models are so complicated, then the first obstacle is finding money to follow the protocols to obtain the right controls*" (researcher)<br><br>"*I think that you can basically make a model of any disease, as long as you have funding to do it*" (HCP)<br><br>**1.6. Technical, equipment and biological**<br>"W*e have many CDG, so we wonder if we need a model for each CDG, and also if we need a model to address each CDG symptom; that's a challenge*" (multiple roles)<br><br>"*Having the whole CNS [central nervous system] manifestations recapitulated in a model is very difficult*" (industry representative)<br><br>"*We're still working to understand what the pathogenesis of CDG is. We presume it's related to glycosylation, but identifying exactly which proteins are glycosylated, what is the underlined effect, particularly for neurologic symptoms we don't know at this point.*" (multiple roles)	**2.5. Financial**<br>"*(…) yeast, worm, and flies (…) those are extremely cheap to make (…) compared to mouse*" (researcher)<br><br>**2.6. Technical, equipment and biological**<br>"*(…) first understanding the pathway mechanism with smaller models (…) and (…) then maybe using AI [artificial intelligence] to look into literature*" (multiple roles)<br>"*You can use a zebrafish, where you can see all of the cells, and you can label the entire development of the organism (…), they're very simple*" (researcher)<br><br>"(…) *glycosylation is very tissue specific, so then iPSC [induced pluripotent stem cells] will be (...) a solution*" (researcher)<br><br>"(…) *there's no invasiveness. These [iPSCs] are obtained from the blood and differentiated into muscle, neuronal cells* (…)" (researcher)<br><br>**2.7. Communication, information-sharing and collaboration/ standardization**<br>"*(…) standardization (...) all the labs will follow the same protocols (…) when using any of these models (…)*" (family member)

Disease models			
**Theme**	**Sub-theme**	**A. CHALLENGES**	**B. SOLUTIONS**
		"(…) we wanted to try to do a CRISPR-Cas9 [gene editing technology] line (...) but that's actually really quite difficult, so it needs really a lot of expertise" (researcher)<br><br>*"You need an aquarium somewhere in your facilities to use it [to work with zebrafish]*" (researcher)<br><br>"*I think for CDG (…) a heterozygous [model] is usually not enough because you don't see any symptoms and a knockout is usually too much, because then you don't have a live animal anymore*" (researcher)	

Table S6. Challenges and solutions of CDG natural history studies (NHS)

Natural history studies (NHS)			
**Theme**	**Sub-theme**	**A. CHALLENGES**	**B. SOLUTIONS**
**Data Management**	Data collection, analysis and quality	**1.1. Administrative/regulatory/ethical**<br>"(...) *when we go to regulatory bodies to design our trials, we find that the endpoints that we need to get approval are not captured in the natural history studies*" (industry representative)<br><br>*"(…) they have to choose some special locations that have more patients now"* (HCP)<br><br>*"(…) but your record-keeping is not the same as what the FDA [Food and Drug Administration] wants, so I got to validate what you do, that's the hardest part"* (industry representative)<br><br>**1.2. Communication, information-sharing and collaboration/ standardization**<br>*"(…) then we do our own, because (...) if we pay for it, and we collect it, we own it, and so then we can say, "no, we are not going to give it to anyone else either"* (industry representative)	**2.1. Technical, equipment and biological**<br>"(…) *make a common dataset where every physician tries to gather on his patients, so (…) we can do a retrospective study, (…) try to do it I think in accordance with the guidelines, which things are really important to look at in the patients (…)"* (multiple roles)<br><br>"*get some attempt at this retrospective natural history for super rare conditions, so if we can enroll a few patients and get an in-depth history from them (...) which I think is hopefully easier now with modern medical records (...)*" (industry representative)<br><br>"*The retrospective studies were great and even bringing artificial intelligence*" (family member)<br><br>*"What about telemedicine as a solution (...) you could have some device or something else (…)"* (researcher)<br><br>*"(...) having the clinician (...) signing in for a Skype consultation with (…) the specialist in CDG, and in that way the natural history can maybe be collected (…)"* (HCP)

Natural history studies (NHS) Theme	Sub-theme	A. CHALLENGES	B. SOLUTIONS
		"(...) *the problem with natural history data is that it has to be valid and to have it valid you need to have someone you trust, someone that does it the way you want it*" (multiple roles)<br><br>**1.3. Financial**<br>"(…) *the natural history studies I can't even imagine how expensive those are*" (family member)<br><br>**1.4. Technical, equipment and biological**<br>"*The number of patients has to be large enough to make conclusions about the natural history [study]*" (multiple roles)	
	Data sharing and accessibility	**1.5. Administrative/regulatory/ethical**<br>"*(...) right now we're in a situation it's not blinded [the NHS] – but we can't share it [the data]" (industry representative*)<br><br>**1.6. Communication, information-sharing and collaboration/standardization**<br>"*they're [NHSs] all collecting the same information (…) because they don't share (…) 80 % of it will be duplicated information*" (industry representative)	**2.2. Communication, information-sharing and collaboration/ standardization**<br>"*we send a letter back to the referring doctor. So, there's always information if we have any publications (…) involved as a consortium. So, it does get around credits (…) the two way [information-sharing] approach is very, very good*" (HCP)
**Patient management**	Patient engagement, recruitment and participation/accessibility	**1.7. Administrative/regulatory/ethical**<br>"*(…) crossing countries within Europe there's different regulations, and in the United States there is different regulations (...) You can send me samples, that is not a problem! It's the patients [being sent to other NHS sites]*" (multiple roles)<br><br>"*(...) just from a logistical perspective (...) in terms of NHS, obviously I don't live in the US (...) to be able to travel that far to take my child for something like that (…) it certainly is something that has stopped me from doing it*" (Family member)<br><br>**1.8. Communication. information-sharing and collaboration/ standardization**	**2.3. Communication, information-sharing and collaboration/ standardization**<br>"*(…) I run CDG UK and all of the families in the UK are there (…) So, I am really the one encouraging them to do that one [the NHS]*" (family member)<br><br>"*(…) we should spread the news better (...) in the CDG newsletter (...) I think it is a good point to translate the newsletter*" (multiple roles)<br><br>"*(...) if the organizations who are taking care of the [natural history] studies (..) think global (…), if a patient is interested in taking part of the study, then this should initiate some sort of action by this organization to get in contact with the (…) local doctor, local clinic*" (family member)

Natural history studies (NHS)			
Theme	Sub-theme	A. CHALLENGES	B. SOLUTIONS
		"(...) share patients with the natural history [studies] (…) we feel that if we send those patients, we are going to lose them" (HCP)<br><br>*"It is disappointing that like my son has a very rare [CDG] and the studies aren't out there for him, and I wish there were"* (family member)<br><br>*"I think that there is a lack of awareness about NHS, for instance no one ever asked us if we want to enter such a program"* (family member)<br><br>**1.9. Financial**<br>*"(…) most of the studies cannot pay for the travel, none of our patients are able to come to NIH [National Institute of Health] (…) it's much more expensive for them, so they don't participate"* (HCP)<br><br>**1.10. Technical expertise, equipment and biological**<br>*"I struggle with the idea of putting him [CDG patient] through all those procedures (...)because I know he would hate it and it would be a horrible time"* (family member)<br><br>*"We did go to the NIH [National Institute of Health] once (…) but my son now has blood clots in his legs, so to fly him there (...) it's just too difficult"* (family member)	*"she [family member] said, I realize that it [participating in a NHS] does not mean that I will have a medication for my child (...) and that maybe I will be only helping future patients (…)"* (HCP)

Table S7. Challenges and solutions of CDG clinical trials (CTs)

Clinical trials (CTs)			
Themes	Sub-themes	A. CHALLENGES	B. SOLUTIONS
**Data Management**	Data collection, analysis and quality	**1.1. Administrative/regulatory/ethical**<br>*"(...) for the clinical trial they have to get approval with the FDA [Food and Drug Administration], and in Europe with EMA [European Medicines Agency] (...) it's a lot of bureaucracy! So, one company may just go to the FDA and not go to the EMA"* (industry representative)<br><br>*"When it's done by normal investigator sites (...) regulators don't accept this kind of research as having enough quality, not because it's done badly,*	**2.1. Communication, information-sharing and collaboration/ standardization**<br>" (…) *finding the right measures [endpoints] and I rely definitely on the patients to give us (…) I've been asking questions along the way (…) always about what are the most important things to you*" (industry representative)<br><br>*"Maybe we could have more awareness of what regulatory agencies are requiring (...) to collect"* (industry representative)

Clinical trials (CTs) Themes	Sub-themes	A. CHALLENGES	B. SOLUTIONS
		just because there hasn't been a third party checking the data along the way" (industry representative)<br><br>**1.2. Financial**<br>*"It is as well a question of costs (...), the cost of doing the investigations (...) some hospitals say no to that"* (HCP)<br><br>**1.3. Technical, equipment and biological**<br>*"It depends on the trial. And it depends as well if the hospital you have locally is able to do all the testing"* (HCP)<br><br>*"(…) And if it is the wrong one [endpoint] and something else gets better but not what they said, it [the trial] fails"* (industry representative)	**2.2. Financial**<br>*"This is a repurposed drug, so the development (…) has been cheap"* (researcher)<br><br>*"(…) clinical trials with the sugar supplementation are not expensive at all"* (industry representative)<br><br>*"Fundraising and cooperation with associations, but I think other solution could be with the countries"* (researcher)<br><br>**2.3. Technical, equipment and biological**<br>*"(…) telemedicine, could be a priority for countries where it's obvious there's no local country specialist, therefore no likelihood for a site"* (researcher)<br><br>*"I like the plan of repurposing drugs. For me it feels like you don't have to reinvent the wheel, you know the side effects. Although it cannot cure, it can help in the management of patients (…)"* (family member)
	Data sharing and accessibility	**1.4. Communication, information-sharing and collaboration/standardization**<br>*"(...) it's difficult to communicate between patient, family, doctor and researcher, because researchers have their own channel to communicate – PubMed (…)"* (family member)<br><br>*"We try to avoid giving negative feedback, so sometimes if we have promising results we share it, if it's negative there's no need to share it with the patients, you know? So, it's confusing to share that (...) we're not good at sharing that"* (multiple roles)	**2.4. Communication, information-sharing and collaboration/ standardization**<br>*"(...) the idea about a website where all the information about trials is, contact details for professionals that are champion in their country (...) that's a good one"* (HCP)<br><br>*"(...) freely share the protocol, so that we all do it the same way, so the data is shareable, we know what labs are monitored, and at what frequency, and what dose (…) and what things are measured in the toxicity"* (HCP)<br>*"(...) you [families] have the strongest voice among all of us (…) sometimes we are faced with logistics and politics, but patients can cross these things"* (HCP)
**Patient managemen t**	Patient engagement, recruitment and participation/acc essibility	**1.5. Communication, information-sharing and collaboration/ standardization**<br>*"For people like us, living in countries where there's no access, it is very hard even to try to access a trial somewhere else"* (family member)	**2.5. Communication, information-sharing and collaboration/ standardization**<br>*"We also need to have the correct consent to say, "do you want feedback?", We need to include that in the consent too"* (multiple roles)

Clinical trials (CTs)			
**Themes**	**Sub-themes**	**A. CHALLENGES**	**B. SOLUTIONS**
		"*(...) most of them [doctors] have never heard of CDG and they're not willing to take part in a clinical trial or monitor us. I tried, but it's just not possible*" (family member)<br><br>"*Because (...) people don't know the trials exist. If people don't know, then they can't volunteer*" (family member)<br><br>"*Sometimes we know there is a clinical trial, but we don't know (…) how long it is, if it's for children or not. We don't have many details*" (family member)<br><br>**1.6. Financial**<br>"*people in different countries will try to participate, depending on our possibilities. It's not something that we can decide unless we have a lot of money for these expenses*" (family member)<br><br>**1.7. Technical expertise, equipment and biological**<br>"*At least for this field of GPI [glycosylphosphatidylinositol] anchor deficiencies there are no available trials (...) so, in some CDG there is even lack of ideas of how to treat patient*s" (HCP)<br><br>"*the experience in Australia is that we don't really have a center at the moment where trials can be run out of* (…)" (family member)<br><br>" *That's the other challenge with rare diseases, there's rare patients, most studies require someone not to be on multiple treatments, so if you're going to be in the Glycomine [Pharmaceutical company looking to advance novel PMM2-CDG treatment] study they don't want you to be on [other investigational therapies]* (...)" (industry representative)	"*I did half [of the paperwork] and then the doctor did the other half (...), and then eventually I had to push it and I've sent it overseas*" (family member)<br><br>"*(…) setting the trial guidelines internationally, that patients could actually (…) in their own country, do the trial*" (family member)<br><br>"*We are enrolled in this CT, but thinking about the future, right? So, this is really important*" (family member)

Table S8. CDG therapy research and development (R&D) tool interdependence.

Challenges	Solutions
**Biobanks and biomarkers**	**Biobanks and Biomarkers**
"*the problem with most rare diseases, especially CDG, is that the numbers of people are so limited and then the numbers of people who participate in the biobanks are so limited that you get a lot of variation (...) without really being able to fully detect patterns [in biomarkers]*" (industry representative)	"(…) *for one person diagnosed with ALG12-CDG (...) we found out the biomarkers. So, it would be very good for us to put this information out somewhere to some biobanks, and to share this information with other scientists*" (researcher)<br><br>"(…) *experts have the access to the facilities they need in order to build that biobank and generate the biomarkers*" (multiple roles)<br><br>"*put together all the samples, because (…) you can compare biomarkers and you can really say that these biomarkers are responsible for this disease, so I think it's very good (…) to have a good access to either blood or fibroblasts*" (HCP)<br><br>"(…) *the larger numbers of patients that we have (...) samples of, the better we can identify any patterns, (...) basically identifying: "is this a real biomarker, is it a helpful biomarker or not?"* (industry representative)
	**Biobanks and disease models**
	"*I think that the biobanks will be the best. We started 3 years ago collecting cells from patients (...) I think that could be a good tool in the future*" (HCP)
	**Biobanks and CTs**
	"(...) *a therapeutic work to come down the pipeline, if that centralized biobank is allowed to tell you [CDG families] (...) That might be an important part of the solution; making a biobank actually useful to going back to the families*" (multiple roles)
**Biomarkers and disease models**	**Biomarkers and Disease models**
"(…) *the available animal models lack changes in transferrin (…) and if the animal model doesn't change that fundamental diagnostic marker, then it brings a lot of doubts*" (industry representative)<br><br>"(…) *one of the issues in CDG is that we don't have any animal model, so we cannot test any drugs, looking at some biomarker that could change, when we actually have a treatment. So that's why it's even worse than in many diseases, because it's difficult to predict what we have to look at to see any change*" (industry representative)	"(…) *making sure we have an animal model, a cell model that recapitulates that [an accessible biomarker] as being fundamental to the disease process, that would be important*" (industry representative)<br><br>"(…) *models are highly necessary for research, for the monitoring or the development of the disease (…) for finding biomarkers*" (researcher)<br><br>"I *think that one of the main insights we have [about CDG pathophysiology] is the involvement of the nervous system in most CDG; hence the allocation of models to identify specific biomarkers for the nervous system*" (researcher)
	**Biomarkers and Patient registries**

**Challenges**	**Solutions**
**Biobanks and biomarkers**	**Biobanks and Biomarkers**
	"I think it will be useful to have (…) patient registries, in which we can fill also the [routinely collected] blood parameters (…)" (HCP)
	**Biomarkers and NHS**
	"(…) finding therapeutic biomarkers (…) is part of what Glycomine is doing with their NHS" (multiple roles)<br><br>"(…) *to get effective biomarkers you need more and more data; so, you can look for correlations across different patients. You can look at longitudinal studies*" (Industry representative)
**Patient registries and CTs**	**Patient registries and CTs**
"I think that the problem is that most of the registries they start like a scientific question, they want to understand the disease, but they are not thinking about therapies at that point" (HCP)	*"(…) the registry then is something that can be used in this type of thing [recruiting patient for clinical trials] (…) think of a registry not run but created by parents, like a global association (…)"* (family member)<br><br>*"(…) our registry needs to think about the disease, the physiopathology of the disease, but also what we can learn about impact in future therapies"* (HCP)
**Biomarkers and CTs**	**Disease models and CTs**
"I know everyone measures transferrin, but we don't know if a drug will impact that or other things in addition" (industry representative)	"(…) *to develop animal models (…) to decide which endpoints of therapies are going to be evaluated, to see if those kinds of therapies are working or not* "(multiple roles)<br><br>*"there's probably others out there that are approved drugs, and if we had the right models, we could reveal that they would work for CDG"* (researcher)
**NHS and CTs**	**NHS and CTs**
"*NIH [National Institute of Health] is not including any nerve conduction in the endpoints [of their NHS], (...) and so for me that's a gap (…) when we go into therapy trials later, if there's no nerve conduction studies, we have to repeat that*" (industry representative)<br><br>"*(…) one of the obstacles could be that only PMM2-CDG has a natural history study (…) because if we want to have a therapeutic clinical trial we have to know about the natural course of the disease*" (HCP)<br><br>*"(…) the regulators are really poorly informed about how to evaluate NHS as comparators [in CTs]"* (Industry representative)	*"(...) figuring out a good primary endpoint that kind of ties into the natural history"* (industry representative)<br><br>"*NHS they address a gap, for rare disease studies (...) if you have a NHS, you don't need to blind this study [the CT]*" (industry representative)

A)

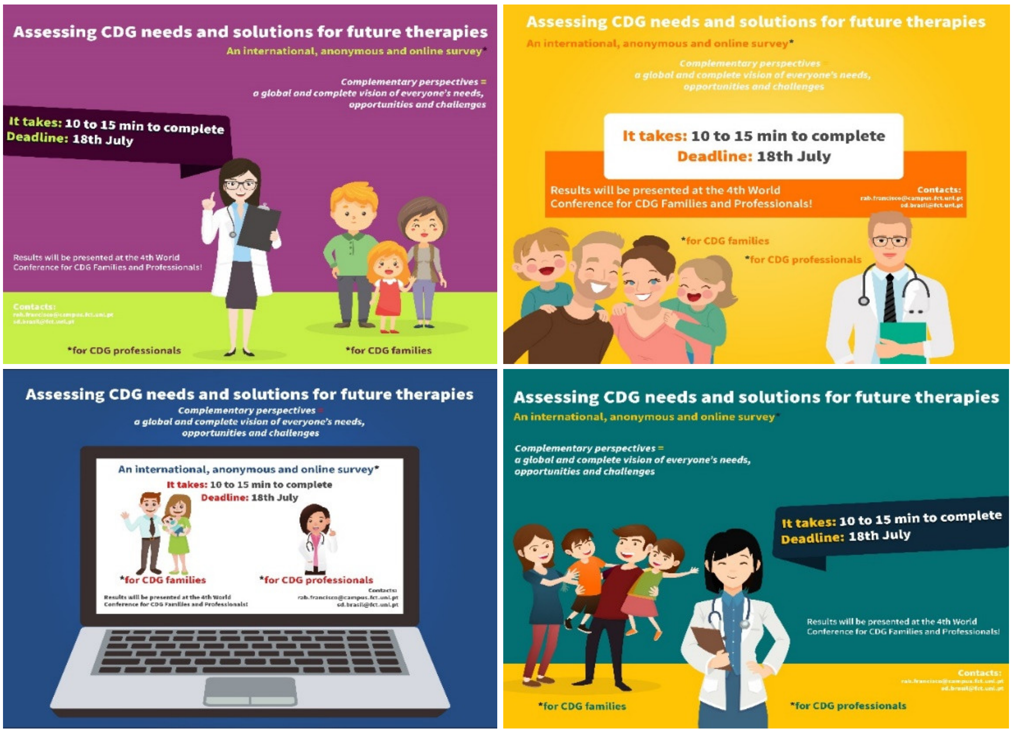

B)

Dear [4th World CDG Conference Participant],

We hope this email finds you well. The 4th World Conference on CDG is right around the corner and we are looking forward to having you.
We are looking forward for this unique networking event that will count with a new format this year. The conference is organized into 8 different workshops with short talks (10 to 15 minutes). Each workshop will be followed by a Think Tank in which the audience will discuss the main topics of the talk (**please read the Power Point file attached**).

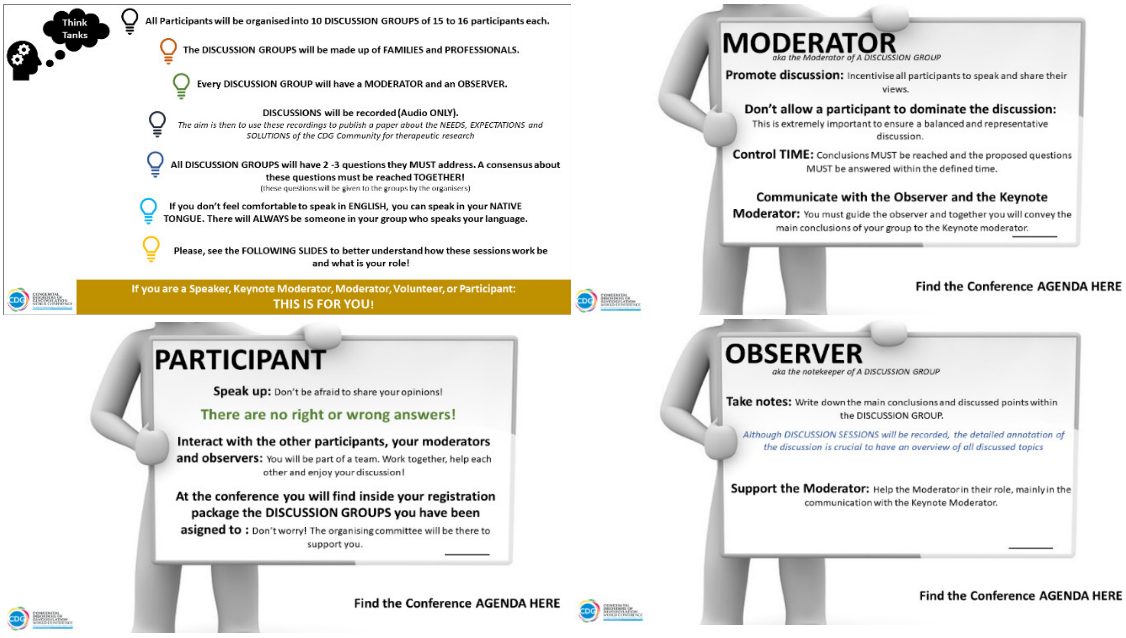

Figure S2. Examples of posts and e-mail messaging used in the online dissemination of the electronic surveys and think tank discussion. A) Posts distributed on social media channels and through emailing; B) Example of an email message with the images of illustrative attached files sent to all the registered participants of the 4th edition of the World Conference on CDG for Families and Professionals.
